# Tree size and relative height interactions enhance generalized additive models for Chinese fir stem taper prediction

**DOI:** 10.48130/forres-0026-0022

**Published:** 2026-06-17

**Authors:** Wanrong Chen, Yanjuan Lyu, Xiangrong Wu, Yuhan Wang, Zongming He, Shuaichao Sun

**Affiliations:** 1 College of Forestry, Fujian Agriculture and Forestry University, Fuzhou, Fujian 350002, China; 2 Engineering Research Center of Chinese Fir, National Forestry and Grassland Administration, Fuzhou, Fujian 350002, China

**Keywords:** Taper model, Smoothing spline function, Main effect, Interaction effect, Relative contribution

## Abstract

Tree stem taper varies dynamically with tree size due to biomechanical constraints governing stability, yet capturing this size-dependent shape plasticity remains a challenge in forest biometrics. Traditional variable-exponent models impose rigid functional forms, while emerging machine learning approaches often obscure these biological relationships within black-box structures. Furthermore, previous applications of generalized additive models (GAMs) have predominantly focused on additive effects, overlooking the critical interactions between tree size and relative position along the stem. To address this gap, we investigated whether explicitly modeling the nonlinear interactions between tree size and relative height could resolve systematic biases in taper prediction. Using a dataset of 516 felled Chinese fir (*Cunninghamia lanceolata*) trees, we constructed GAMs incorporating tensor product interaction smooths and benchmarked them against the widely used parametric models and two machine learning algorithms. We found that GAMs relying solely on main effects failed to outperform the parametric benchmark. However, introducing explicit interaction terms, specifically between diameter at breast height and relative height, substantially reduced the root mean square error by 52.3% compared to the additive GAM and surpassed both the established Kozak-II benchmark and the optimized machine learning models in validation accuracy. Variable importance analysis confirmed that these interactions act as critical modifiers that drive the model improvement by accurately capturing the ontogenetic drift in stem form, such as the pronounced basal flare in larger trees. These findings demonstrate that interaction-inclusive GAMs serve as a powerful tool to bridge the trade-off between algorithmic flexibility and parametric interpretability, offering a biologically grounded approach for precision forest inventory.

## Introduction

Sustainable forest management and the precise estimation of carbon stocks rely fundamentally on the accurate quantification of timber resources^[1,2]^. While stem taper models serve as indispensable tools for these tasks by describing the gradual decrease in stem diameter with increasing height^[3,4]^, stem taper is not a static geometric attribute. Instead, it represents a dynamic phenotype that evolves with tree ontogeny. According to the constant stress hypothesis and metabolic scaling theories, trees allocate biomass along the stem to equalize the distribution of bending stress caused by wind loading and self-weight^[5−8]^. As a tree matures and increases in size, its stem form factor must evolve to maintain stability. For instance, larger dominant trees typically develop a more pronounced basal flare to resist higher bending moments compared to smaller subdominant individuals^[9]^. This allometric scaling implies that the taper profile is inextricably dependent on the absolute size of the tree. Therefore, accurate taper models must account for this nonlinear interplay between tree size and relative position along the stem to capture the inherent biological plasticity of tree form.

The quest for taper modeling boasts a rich history spanning over a century with seminal contributions from researchers like Larson^[5]^, Newnham^[10]^, and notably Kozak^[11,12]^, whose variable-exponent models represent a widely adopted benchmark in forest inventory^[13−17]^. These traditional parametric approaches possess inherent advantages regarding interpretability as their parameters often carry direct biological meanings that can be incorporated into hierarchical modeling frameworks^[18−20]^. However, their reliance on predefined mathematical forms imposes a limitation. Specifically, such structural constraints can restrict flexibility in capturing the inherent variability and complex shapes of tree stems, especially under diverse site conditions or for trees exhibiting irregular growth patterns^[21,22]^. This inherent rigidity has motivated the exploration of more adaptable modeling frameworks.

Generalized additive models (GAMs) offer a compelling alternative by blending the interpretability of linear models with the flexibility of nonparametric smoothing functions to capture complex data-driven relationships without stringent *a priori* assumptions^[23,24]^. While pioneering studies^[21,22,25,26]^ successfully established the viability of GAMs in taper modeling, they primarily focused on capturing the additive effects of key predictors independently. This additive assumption overlooks the critical interaction effects where the influence of relative height on taper varies significantly depending on tree size. Although recent machine learning algorithms like artificial neural networks (ANN) and random forest (RF) can effectively capture such complex nonlinearities^[22,27]^, these methods often operate as black boxes that prioritize predictive accuracy at the expense of biological interpretability^[28,29]^. Consequently, there is a lack of modeling frameworks that can bridge this gap by offering the predictive power of machine learning regarding interaction effects while retaining the inferential transparency of parametric models. While GAMs have shown promise, the application of interaction-inclusive models utilizing tensor product smooths^[30]^ to resolve this modeling trade-off remains underexplored.

In this study, we address this knowledge gap using Chinese fir (*Cunninghamia lanceolata*) as a model species. As the most extensively planted species in Southern China with paramount economic and ecological significance^[31−33]^, Chinese fir exhibits rapid growth and significant morphological variation, making it an ideal candidate for examining size-dependent taper dynamics. We address the limitations of previous methods by developing GAM-based taper models that explicitly incorporate interaction effects of varying complexity. Our primary objective is to construct GAMs incorporating tensor product interactions to identify the optimal structure for stem taper prediction. We subsequently evaluate the performance of these models against established parametric equations and popular machine learning algorithms while quantifying the relative contributions of main and interactive effects. Ultimately, we aim to connect these statistical interaction patterns with the biological principles governing size-dependent stem form development.

## Materials and methods

### Data

The stem form data for this study were collected from Fujian Province in Southeastern China, a region renowned for its production of high-quality Chinese fir timber. The province is characterized by a high forest coverage rate of 65.12%, an extensive mountainous landscape, and a humid, rainy climate throughout the year. These environmental conditions provide an ideal natural habitat for the robust growth of Chinese fir.

**Figure 1:**
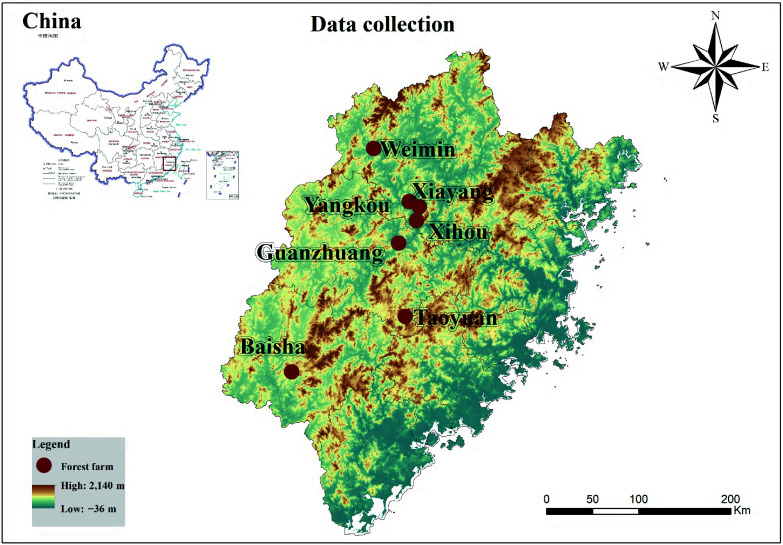
Geographic locations of the seven state-owned forest farms in Fujian Province, China, where sample data were collected.

Data were sourced from seven state-owned forest farms strategically located across the province including Guanzhuang (117°35'−117°50' E, 26°24'−27°38' N), Taoyuan (117°32'−117°48' E, 25°15'−25°22' N), Baisha (116°30'−116°38' E, 25°04'−25°15' N), Xihou (118°02'−118°13' E, 26°36'−26°47' N), Yangkou (117°46'−118°25' E, 26°28'−26°54' N), Weimin (117°48'−117°51' E, 26°56'−26°57' N), and Xiayang (117°54'−118°02' E, 26°44'−26°52' N). Figure 1 shows the specific locations of each forest farm.

**Figure 2:**
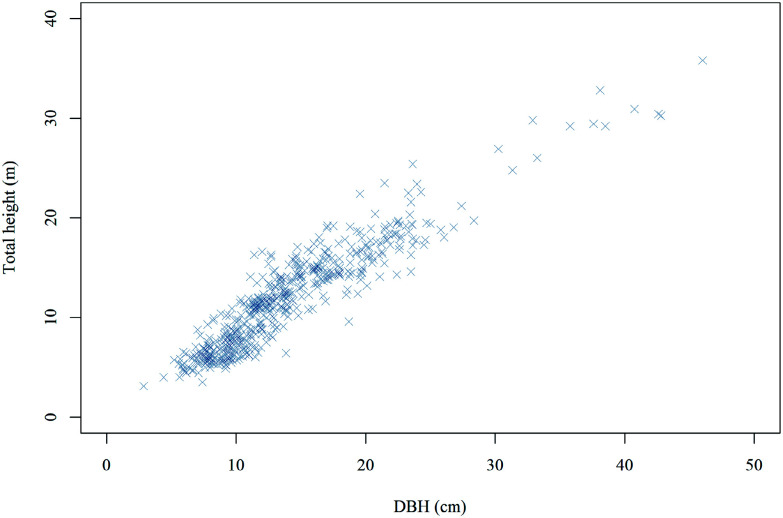
Height-DBH distribution of the sampled Chinese fir trees. DBH denotes diameter at breast height.

For each sample tree felled, the tree age and an identification number were recorded. The DBH and total tree height (*H*) were then measured. Following this, the stem diameter was systematically measured at multiple predetermined positions along the bole starting from the base (0 m) and continuing at relative heights of 0.02, 0.04, 0.06, 0.08, 0.1, 0.15, 0.2, 0.25, 0.3, 0.4, 0.5, 0.6, 0.7, 0.75, 0.8, 0.9, and 0.95 *H*. In total, 516 felled Chinese fir trees were measured, yielding 10,374 individual diameter observations. Summary statistics for these measurements are presented in Table 1, while Fig. 2 illustrates the height-DBH distribution for the entire dataset.

**Table 1:** Summary statistics for the sampled Chinese fir trees.

Variable	Minimum	Maximum	Mean	SD	CV (%)
*D* (cm)	2.86	46.01	15.57	5.96	35.46
*H* (m)	3.13	35.80	13.34	4.85	23.50
*d* (cm)	0.27	63.16	11.37	6.12	37.42
*h* (m)	0.00	32.22	5.99	4.75	22.52
*D* is diameter at breast height; *H* is total tree height; *d* is stem diameter at height *h*; *h* is stem height from the butt; SD is standard deviation, CV is coefficient of variation.					

### Conventional parametric taper models

To identify a benchmark for performance, we first fitted the Chinese fir stem profile data using four widely recognized variable-exponent taper equations. These models, selected for their frequent application and proven utility in forest inventory, were the Zeng & Liao^[34]^ model, the Lee^[35]^ model, and two variants of the Kozak^[12]^ model, herein referred to as Kozak-I and Kozak-II. The mathematical forms of these models are as follows:

1 $ d=D{\left(\dfrac{H-h}{H-1.3}\right)}^{{{a}_{0}}+{{a}_{1}}{{\left(\tfrac{h}{H}\right)}^{\tfrac{1}{4}}}+{{a}_{2}}{{\left(\tfrac{h}{H}\right)}^{\tfrac{1}{2}}}+{{a}_{3}}\left(\tfrac{D}{H}\right)}+\varepsilon $

2 $ d={a}_{0}{D}^{{{a}_{1}}}{\left(1-\dfrac{h}{H}\right)}^{{{a}_{2}}{{\left(\tfrac{h}{H}\right)}^{2}}+{{a}_{3}}\left(\tfrac{h}{H}\right)+{{a}_{4}}}+\varepsilon $

3 $d={a}_{0}{D}^{{{a}_{1}}}{\left(\dfrac{1-{\left(\dfrac{h}{H}\right)}^{\tfrac{1}{4}}}{1-0.0{1}^{\tfrac{1}{4}}}\right)}^{\left[{a}_{2}+{a}_{3}\left(\tfrac{1}{\exp⁡\left(\tfrac{D}{H}\right)}\right)+{a}_{4}D\left(\tfrac{1-{\left(\tfrac{h}{H}\right)}^{\tfrac{1}{4}}}{1-0.0{1}^{\tfrac{1}{4}}}\right)+{a}_{5}{\left(\tfrac{1-{\left(\tfrac{h}{H}\right)}^{\tfrac{1}{4}}}{1-0.0{1}^{\tfrac{1}{4}}}\right)}^{\tfrac{D}{H}}\right]}+\varepsilon $

4 $ d={a}_{0}{D}^{{{a}_{1}}}{H}^{{{a}_{2}}}{\left[\dfrac{1-{\left(\dfrac{h}{H}\right)}^{\tfrac{1}{3}}}{1-{\left(\dfrac{1.3}{H}\right)}^{\tfrac{1}{3}}}\right]}^{\left[{a}_{3}{\left(\tfrac{h}{H}\right)}^{4}+{a}_{4}\left(\tfrac{1}{\exp⁡\left(\tfrac{D}{H}\right)}\right)+{a}_{5}{\left(\tfrac{1-{\left(\tfrac{h}{H}\right)}^{\tfrac{1}{3}}}{1-{\left(\tfrac{1.3}{H}\right)}^{\tfrac{1}{3}}}\right)}^{0.1}+{a}_{6}\left(\tfrac{1}{D}\right)+{a}_{7}{H}^{1-{{\left(\tfrac{h}{H}\right)}^{\tfrac{1}{3}}}}+{a}_{8}\left(\tfrac{1-{\left(\tfrac{h}{H}\right)}^{\tfrac{1}{3}}}{1-{\left(\tfrac{1.3}{H}\right)}^{\tfrac{1}{3}}}\right)\right]}+\varepsilon $

where, *d* represents the diameter at various stem positions (cm); *h* denotes the height at those positions (m); *D* is the diameter at breast height (DBH, cm); *H* indicates total tree height (m); *h*/*H* signifies relative height; *a*_0_, *a*_1_, *a*_2_, *a*_3_, *a*_4_, *a*_5_, *a*_6_, *a*_7_, and *a*_8_ are model parameters; and *ε* is the random error term.

### Generalized additive taper models

The fundamental mathematical expression for a generalized additive model (GAM) is:

5 $ Y=\alpha +{s}_{1}\left({x}_{1}\right)+{s}_{2}\left({x}_{2}\right)+\cdots +{s}_{m}\left({x}_{m}\right)+\epsilon $

where, *Y* is the response variable; *α* is the intercept; *x*_1_, …, *x*_*m*_ are the explanatory variables; *s*(·) denotes a nonparametric smoothing spline function for each variable; and *m* is the number of predictors.

To ensure a fair and direct comparison with the parametric models, we maintained the same variable selection in developing the GAM-based taper models. We designated the stem diameter at a given height (*d*) as the response variable, with DBH (*D*), total tree height (*H*), and relative height (*h*/*H*) serving as the explanatory variables. To determine the most appropriate model structure, we formulated and compared four distinct GAMs based on different variable transformations^[36]^:

6 $ d=\alpha +{s}_{1}\left(D\right)+{s}_{2}\left(H\right)+{s}_{3}\left(h/H\right) $

7 $ d=\alpha +{s}_{1}\left({D}^{2}\right)+{s}_{2}\left(H\right)+{s}_{3}\left(h/H\right) $

8 $ d=\alpha +{s}_{1}\left(D\right)+{s}_{2}\left(H\right)+{s}_{3}\left(\sqrt{h/H}\right) $

9 $ d=\alpha +{s}_{1}\left({D}^{2}\right)+{s}_{2}\left(H\right)+{s}_{3}\left(\sqrt{h/H}\right) $

To examine the influence of different spline functions on GAM performance, we constructed models using seven distinct types of splines after identifying the optimal model structure from Eqs. (6)−(9). The evaluated splines included basis spline (BS), penalized B-spline (PS), Duchon spline (DS), thin plate spline (TP), Gaussian process spline (GP), cubic regression spline (CR), and cubic cyclic spline (CC). All GAMs were fitted using the restricted maximum-likelihood (REML) method. This approach was chosen over standard maximum likelihood (ML) to mitigate the potential for downward bias in variance component estimation, thereby enhancing model stability.

A key strength of GAMs lies in their capacity to effectively model complex, nonlinear interactions between variables. We explored these interactions using tensor product interaction smooths, which allow for the explicit separation of interaction effects from the main effects of the predictors. To identify the optimal trade-off between model complexity and predictive power, we constructed a series of candidate GAMs incorporating different combinations of interaction terms based on the optimal main-effect structure. Taking Eq. (6) as a base example, a GAM incorporating all two-way interactions would take the following form:

10 $ d=\alpha +{s}_{1}\left(D\right)+{s}_{2}\left(H\right)+{s}_{3}\left(h/H\right)+t{i}_{1}\left(D,H\right)+t{i}_{2}\left(D,h/H\right)+t{i}_{3}\left(H,h/H\right) $

where, *ti*(·) denotes the tensor product interaction smooth function.

### Machine learning models

To provide a comprehensive benchmark for the proposed GAMs, we developed two widely used machine learning models: artificial neural networks (ANN) and random forest (RF). Both models utilized the same predictors as the GAMs (*D*, *H*, and relative height terms) to ensure comparability.

The ANN model was constructed using a multilayer perceptron architecture with the neuralnet package in R^[37]^. We employed a resilient backpropagation algorithm with a single hidden layer. To determine the optimal network architecture, we performed a grid search for the number of neurons in the hidden layer (ranging from 1 to 10), selecting the configuration that minimized the mean square error in the cross-validation. Based on this grid search, the optimal network architecture was established as a single hidden layer with three neurons.

The RF model was implemented using the randomForest package^[38]^. We optimized two key hyperparameters: the number of trees (ntree) and the number of variables randomly sampled at each split (mtry). The optimal hyperparameter combination was identified by evaluating the out-of-bag error and cross-validation performance across a range of candidate values. Consequently, the final hyperparameters were set to 1,000 for the number of trees and 2 for the number of variables randomly sampled at each split.

### Model evaluation

The performance of all taper models was evaluated using a five-fold cross-validation procedure based on tree ID. The entire dataset of 516 trees was randomly partitioned into five mutually exclusive subsets. In an iterative process, four subsets were combined for model training, and the remaining one was used for validation. This process was repeated five times until every tree had served as a validation sample exactly once. The final model performance was assessed by averaging the evaluation metrics from the five validation folds.

To ensure statistical validity, the selection of evaluation criteria depended on the specific modeling context. We employed Akaike's information criterion (AIC), Bayesian information criterion (BIC), and −2*log-likelihood (−2LogLik) exclusively for the internal performance assessment of the four candidate parametric models. Lower values for these indices signify a superior fit. For all other model evaluations, we relied on standard predictive metrics for both model fitting and cross-validation. These metrics comprised the root mean square error (RMSE), mean absolute error (MAE), mean absolute percentage error (MAPE), and fit index (*R*^2^). Superior predictive performance is indicated by lower values of RMSE, MAE, and MAPE, and a higher value of *R*^2^. The formulas for these statistical indicators are as follows:

11 $ {\mathrm{AIC}}=-2\ln (L)+2k $

12 $ {\mathrm{BIC}}=-2\ln (L)+k\ln (n) $

13 $ {\mathrm{RMSE}}=\sqrt{\dfrac{1}{n}\sum\limits_{i=1}^{n}{\left({d}_{i}-{\hat{d}}_{i}\right)}^{2}} $

14 $ {\mathrm{MAE}}=\dfrac{1}{n}\sum_{i=1}^{n}\left| {d}_{i}-{\hat{d}}_{i}\right| $

15 $ {\mathrm{MAPE}}=\dfrac{100{\%} }{n}\sum\limits_{i=1}^{n}\left| \dfrac{{d}_{i}-{\hat{d}}_{i}}{{d}_{i}}\right| $

16 $ {R}^{2}=1-\dfrac{\displaystyle\sum\limits_{i=1}^{n}{\left({d}_{i}-{\hat{d}}_{i}\right)}^{2}}{\displaystyle\sum\limits_{i=1}^{n}{\left({d}_{i}-\overline{d}\right)}^{2}} $

where, *k* is the number of model parameters; *n* is the sample size; *L* is the likelihood function; *d*_*i*_ and $ {\hat{d}}_{i} $ are the *i*^th^ observed and predicted stem diameter; and $ \bar{d} $ is the mean of the observed stem diameters.

### Variable importance analysis

Based on the optimal GAM taper equation, the relative importance of each predictor and their interaction terms was quantified using the gam.hp package in R^[39]^. This approach implements hierarchical partitioning to decompose the total explained deviance into components attributable to each predictor's independent contribution, thereby providing robust importance estimates even in the presence of multicollinearity.

## Results

### Comparison of parametric benchmarks and main-effect GAMs

Our baseline analysis demonstrated that conventional parametric models establish a stringent benchmark for predictive accuracy. Among the four variable-exponent candidates (M1−M4), the Kozak-II model (M4) demonstrated the superior goodness-of-fit, yielding the lowest AIC, BIC, and −2LogLik values (Table 2). In contrast, generalized additive models constructed solely with main effects proved insufficient to capture the full complexity of taper dynamics. Although the screening of predictor functional forms (Table 3) and smoothing splines (Table 4) identified the Duchon spline model incorporating *D*, *H,* and $ \sqrt{h/H} $ (M11) as the optimal additive structure, it failed to surpass the predictive accuracy of the parametric benchmark (Table 5).

**Table 2:** Goodness-of-fit comparison of conventional parametric taper models for Chinese fir.

No.	Model	AIC	BIC	−2LogLik
M1	Zeng and Liao^[34]^	29,709.38	29,745.62	29,699.38
M2	Lee^[35]^	30,270.91	30,314.39	30,258.91
M3	Kozak-I^[12]^	31,084.23	31,134.96	31,070.23
M4	Kozak-II^[12]^	**26,782.29**	**26,854.76**	**26,762.29**
Bold numbers denote the best model for each criterion.				

**Table 3:** Comparison of goodness-of-fit statistics for main-effect GAMs using different variable transformations.

No.	Model	RMSE	MAE	MAPE	*R* ^2^
M5	$ d=\alpha +{s}_{1}\left(D\right)+{s}_{2}\left(H\right)+{s}_{3}\left(h/H\right) $	1.8017	1.1957	**19.3549**	0.9132
M6	$ d=\alpha +{s}_{1}\left({D}^{2}\right)+{s}_{2}\left(H\right)+{s}_{3}\left(h/H\right) $	1.8033	1.1960	19.3744	0.9131
M7	$ d=\alpha +{s}_{1}\left(D\right)+{s}_{2}\left(H\right)+{s}_{3}(\sqrt{h/H}) $	**1.7814**	**1.1798**	19.3742	**0.9152**
M8	$ d=\alpha +{s}_{1}\left({D}^{2}\right)+{s}_{2}\left(H\right)+{s}_{3}(\sqrt{h/H}) $	1.7830	1.1806	19.3996	0.9150
Bold numbers denote the best model for each criterion.					

**Table 4:** Comparison of goodness-of-fit statistics for main-effect GAMs constructed with different smoothing spline functions.

No.	Spline function	RMSE	MAE	MAPE	*R* ^2^
M9	BS	1.7830	1.1793	19.3216	0.9150
M10	PS	1.7831	1.1794	19.3263	0.9150
M11	DS	**1.7789**	**1.1768**	**19.3203**	**0.9154**
M12	TP	1.7814	1.1798	19.3742	0.9152
M13	GP	1.7804	1.1789	19.3737	0.9153
M14	CR	1.7825	1.1797	19.3603	0.9151
M15	CC	2.9298	1.7361	31.5170	0.7706
Bold numbers denote the best model for each criterion.					

**Table 5:** Cross-validation statistics for parametric and main-effect additive taper models.

No.	RMSE	MAE	MAPE	*R* ^2^
M1	1.0192	0.6340	7.6203	0.9719
M2	1.0456	0.7345	8.6664	0.9704
M3	1.0866	0.7955	10.9481	0.9681
M4	**0.8746**	**0.5828**	**7.1727**	**0.9793**
M9	1.8058	1.1945	19.5991	0.9127
M10	1.8065	1.1952	19.6147	0.9126
M11	1.8045	1.1945	19.5934	0.9130
M12	1.8027	1.1954	19.6199	0.9130
M13	1.8021	1.1945	19.6288	0.9130
M14	1.8016	1.1945	19.6139	0.9131
M15	3.0705	1.7842	32.0782	0.7421
Bold numbers denote the best model for each criterion.				

**Figure 3:**
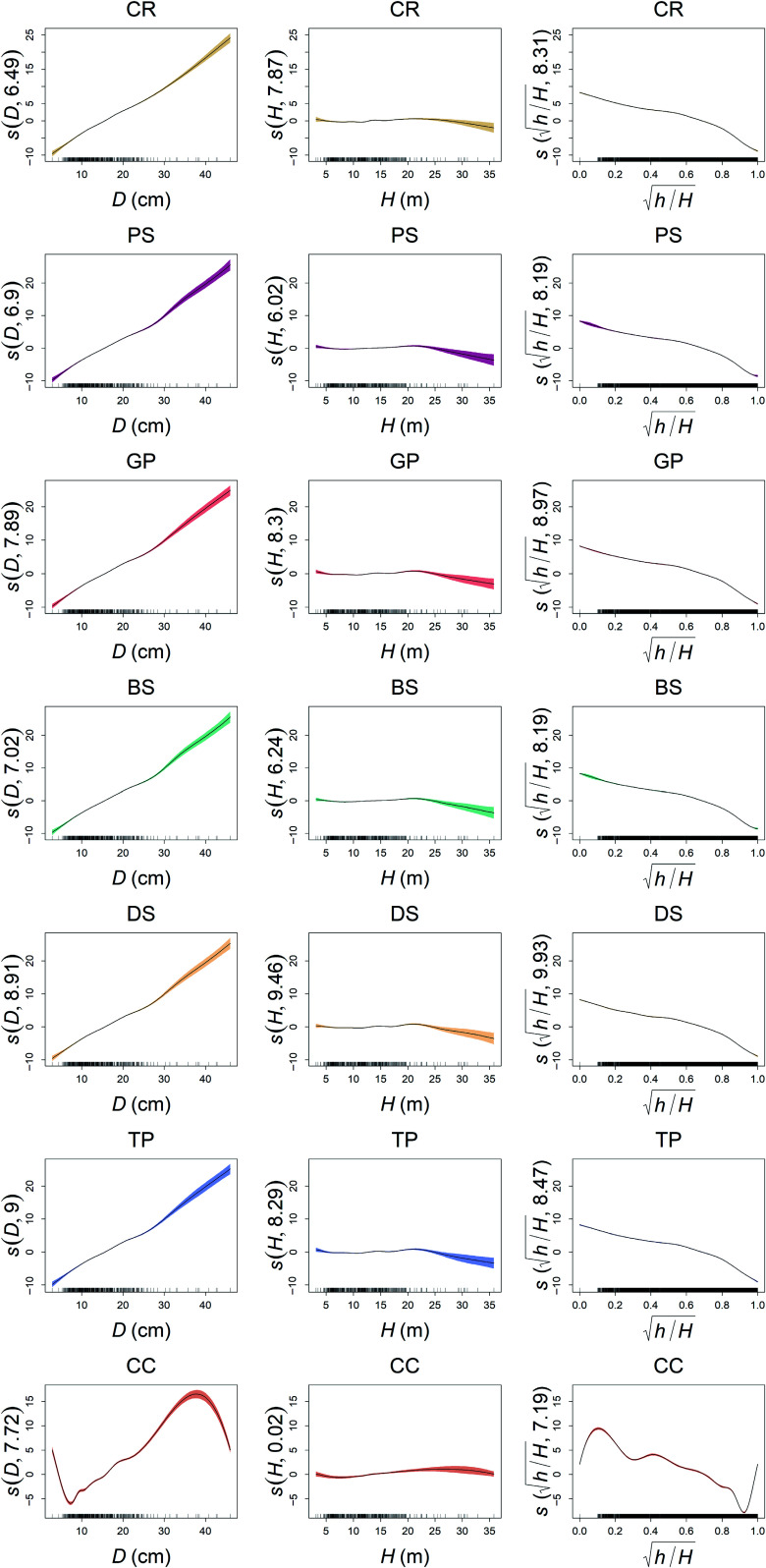
Partial effects of variables for different smoothing spline functions. The numbers in parentheses on the *Y*-axis labels represent the estimated degrees of freedom (edf) for each smooth term, indicating the complexity of the fitted nonlinear curve.

The partial effects of the predictors derived from these main-effect GAMs are visualized in Fig. 3. Consistent patterns emerged across all spline types except CC. Specifically, the predicted stem diameter (*d*) exhibited a gradual nonlinear increase with *D* alongside widening confidence intervals. The relationship between *d* and *H* remained relatively stable but demonstrated greater uncertainty at larger heights. Additionally, *d* displayed a pronounced nonlinear decline with increasing $ \sqrt{h/H} $ while maintaining narrow and stable confidence intervals.

### Performance enhancement via interaction terms

Explicitly modeling tensor product interactions overcame the structural limitations of additive frameworks and yielded significant performance gains. Seventy-seven GAMs with distinct two-way and three-way interaction structures across various spline functions were examined, and the four best-performing GAMs representing varying levels of interaction complexity are presented in Table 6. The inclusion of interaction terms substantially reduced fitting errors, where the model incorporating all two-way interactions (M18) achieved the highest fitting performance. Meanwhile, the model with two interactions (M17 excluding the *D* and *H* interaction) demonstrated comparable accuracy.

**Table 6:** Performance comparison of the best-performing GAMs with varying levels of interaction complexity.

No.	Model	Spline function	RMSE	MAE	MAPE	*R* ^2^
M16	$ d=\alpha +{s}_{1}\left(D\right)+{s}_{2}\left(H\right)+{s}_{3}\left(\sqrt{h/H}\right)+{ti}_{1}\left(D,\sqrt{h/H}\right) $	GP	0.8534	0.5776	7.2471	0.9805
M17	$ d=\alpha +{s}_{1}\left(D\right)+{s}_{2}\left(H\right)+{s}_{3}\left(\sqrt{h/H}\right)+{ti}_{1}(D,\sqrt{h/H})+{ti}_{2}(H,\sqrt{h/H}) $	DS	0.8212	0.5404	**6.6636**	0.9820
M18	$ d=\alpha +{s}_{1}\left(D\right)+{s}_{2}\left(H\right)+{s}_{3}\left(\sqrt{h/H}\right)+{ti}_{1}\left(D,H\right)+{ti}_{2}\left(D,\sqrt{h/H}\right)+{ti}_{3}(H,\sqrt{h/H}) $	BS	**0.8129**	**0.5365**	6.6722	**0.9823**
M19	$ d=\alpha +{s}_{1}\left(D\right)+{s}_{2}\left(H\right)+{s}_{3}\left(\sqrt{h/H}\right)+{ti}_{1}\left(D,H,\sqrt{h/H}\right) $	BS	0.8316	0.5512	6.8612	0.9815
Bold numbers denote the best model for each criterion.						

However, the five-fold cross-validation results summarized in Table 7 highlighted a critical trade-off between complexity and generalization. The more parsimonious model (M17), which incorporates only two specific interaction terms $ {ti}_{1}(D,\sqrt{h/H}) $ and $ {ti}_{2}(H,\sqrt{h/H}) $, demonstrated the best generalization capability among all representative models. M17 yielded the lowest validation RMSE (0.8688 cm), MAE (0.5765 cm), MAPE (7.05%), and the highest *R*^2^ (0.9796), outperforming both the complex M18 and the parametric benchmark (M4). Crucially, this interaction-inclusive GAM (M17) also demonstrated superior predictive power compared to the black-box machine learning algorithms, surpassing both the artificial neural network (M20) and random forest (M21) benchmarks.

**Table 7:** Cross-validation performance metrics comparing the interaction-inclusive GAMs against parametric and machine learning benchmarks.

No.	Model	RMSE	MAE	MAPE	*R* ^2^
M4	Kozak-II	0.8746	0.5828	7.1727	0.9793
M11	$ d=\alpha +{s}_{1}\left(D\right)+{s}_{2}\left(H\right)+{s}_{3}(\sqrt{h/H}) $	1.8045	1.1956	19.5934	0.9128
M16	$ d=\alpha +{s}_{1}\left(D\right)+{s}_{2}\left(H\right)+{s}_{3}\left(\sqrt{h/H}\right)+{ti}_{1}\left(D,\sqrt{h/H}\right) $	0.8864	0.6003	7.5216	0.9787
M17	$ d=\alpha +{s}_{1}\left(D\right)+{s}_{2}\left(H\right)+{s}_{3}\left(\sqrt{h/H}\right)+{ti}_{1}(D,\sqrt{h/H})+{ti}_{2}(H,\sqrt{h/H}) $	**0.8688**	**0.5765**	**7.0451**	**0.9796**
M18	$ d=\alpha +{s}_{1}\left(D\right)+{s}_{2}\left(H\right)+{s}_{3}\left(\sqrt{h/H}\right)+{ti}_{1}\left(D,H\right)+{ti}_{2}\left(D,\sqrt{h/H}\right)+{ti}_{3}(H,\sqrt{h/H}) $	0.8965	0.5791	7.0870	0.9783
M19	$ d=\alpha +{s}_{1}\left(D\right)+{s}_{2}\left(H\right)+{s}_{3}\left(\sqrt{h/H}\right)+{ti}_{1}\left(D,H,\sqrt{h/H}\right) $	1.2807	0.6148	7.4740	0.9441
M20	Artificial Neural Networks (ANN)	0.8742	0.5836	7.2455	0.9793
M21	Random Forest (RF)	1.1009	0.7289	9.3892	0.9673
Bold numbers denote the best model for each criterion.					

The detailed parameter statistics based on the entire dataset for the optimal M17 model are provided in Table 8. As shown, all main effect and interaction smooth terms exhibited estimated degrees of freedom (edf) substantially greater than 1. This statistically confirms the highly nonlinear nature of these biological relationships, with higher edf values reflecting the complex, flexible curve structures required to accurately model the taper profile.

**Table 8:** Parameter estimates and smooth term statistics of the optimal interaction-inclusive GAM (M17).

Term	Estimate (SE)	edf	Ref.df	*p*-value
Intercept	11.458 (0.008)			< 0.001
$ {s}_{1}(D) $		9.593	10.57	< 0.001
$ {s}_{2}(H) $		10.660	10.95	< 0.001
$ {s}_{3}(\sqrt{h/H}) $		10.557	10.95	< 0.001
$ {ti}_{1}(D,\sqrt{h/H}) $		13.454	14.87	< 0.001
$ {ti}_{2}(H,\sqrt{h/H}) $		13.955	15.32	< 0.001
SE denotes standard error; edf and Ref.df are effective degrees of freedom and reference degrees of freedom respectively.				

### Statistical interpretation and bias correction

**Figure 4:**
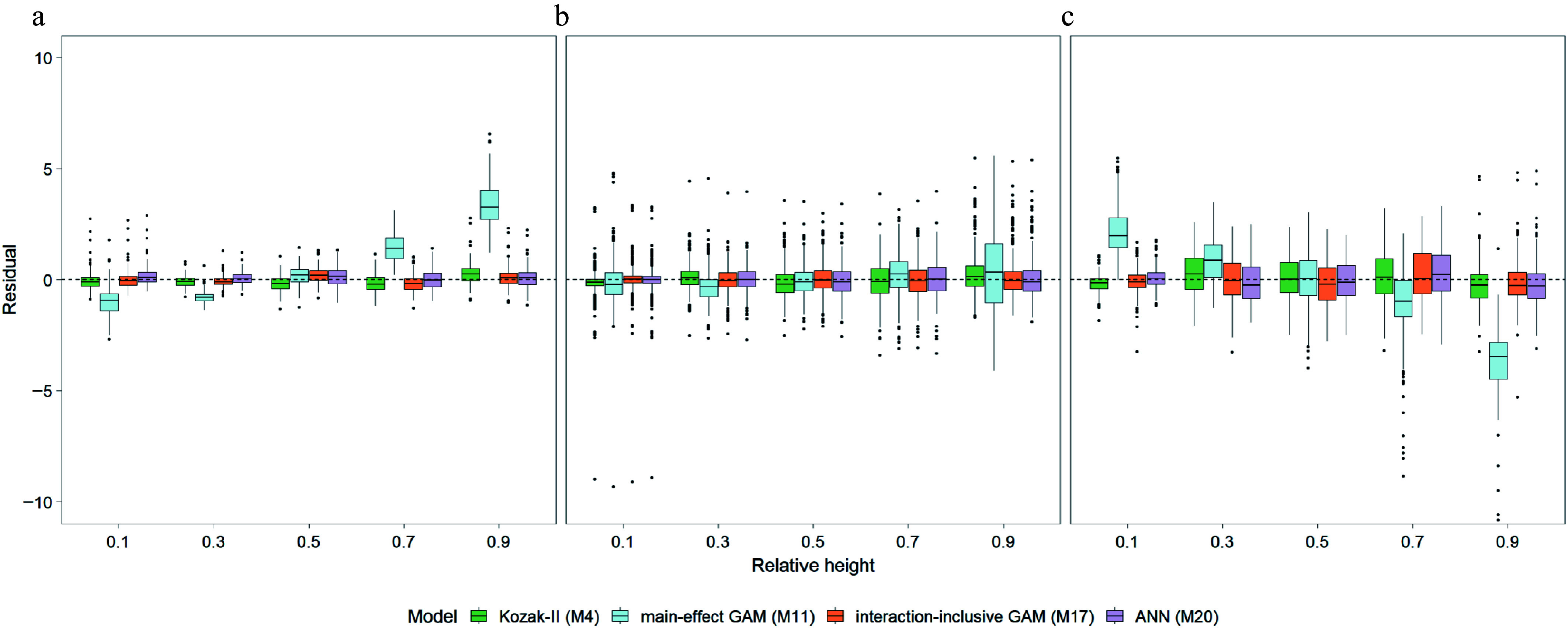
Boxplots of prediction residuals across relative stem heights for (a) small (*D* < 10 cm), (b) medium (10 cm ≤ *D* < 20 cm), and (c) large trees (*D* ≥ 20 cm). The comparison includes the benchmark Kozak-II model (M4), the best main-effect GAM (M11), the optimal interaction-inclusive GAM (M17), and the ANN model (M20).

Stratified residual analysis (Fig. 4) elucidated the source of this predictive superiority. The main-effect GAM (M11) exhibited systematic bias characterized by the overestimation of top diameters in small trees (*D* < 10 cm) and underestimation in large trees (*D* ≥ 20 cm). In contrast, the optimal interaction-inclusive model (M17) effectively corrected these size-dependent structural deviations, maintaining homogeneous residuals centered near zero. Notably, M17 demonstrated superior stability with slightly tighter residual distributions compared to both the benchmark Kozak-II model (M4) and the ANN model (M20), confirming its robust predictive capability across all tree sizes and stem heights.

**Figure 5:**
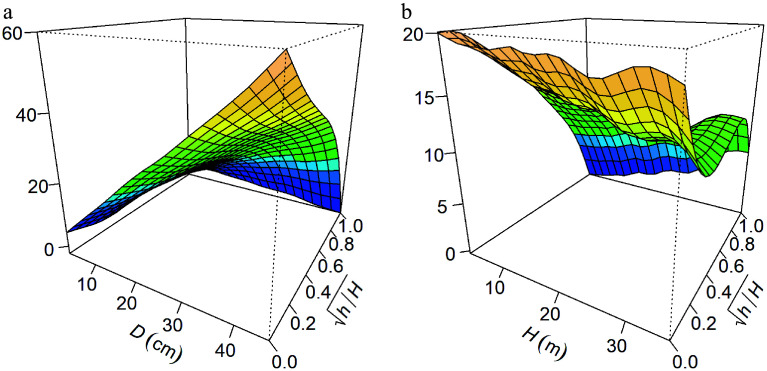
Visualization of the tensor product interaction effects on predicted stem diameter from the optimal GAM (M17). The plots show the interactions between (a) *D* and $ \sqrt{h/H} $, and (b) *H* and $ \sqrt{h/H} $. The warping of the surface plane indicates the magnitude of the interaction effect.

The statistical basis for this correction is illustrated in the three-dimensional visualization of tensor product surfaces (Fig. 5). The interaction between DBH and $ \sqrt{h/H} $ manifested as a steep, nonlinear surge in predicted diameter at the stem base specifically for large-diameter trees (Fig. 5a), accurately capturing the biological phenomenon of basal flare. Meanwhile, the interaction between total height and $ \sqrt{h/H} $ (Fig. 5b) was primarily concentrated in the lower stem section across the entire range of tree heights. Unlike the dominant DBH interaction, this surface displayed a significantly lower *z*-axis range and a more subtle curvature, indicating that total height exerts a continuous but moderate influence on the stem taper profile, serving as a modifying factor rather than a primary driver of diameter magnitude.

**Figure 6:**
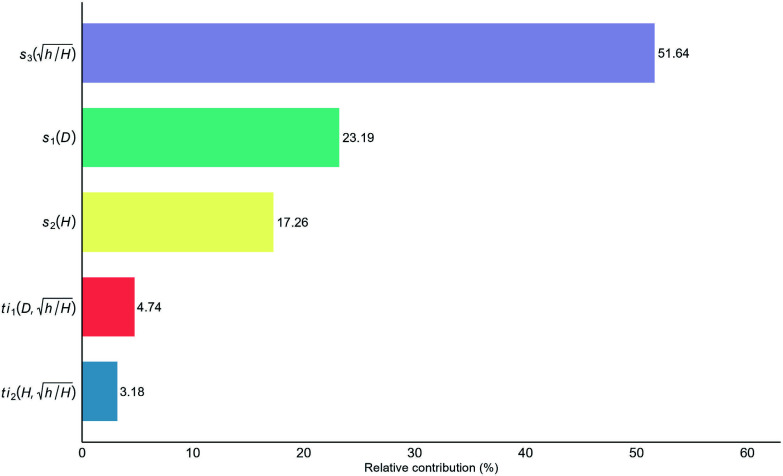
Relative importance of predictors in the optimal interaction-inclusive GAM (M17), quantified as the percentage contribution of each main effect and interaction term to the total explained deviance.

Variable importance analysis (Fig. 6) quantified this architectural hierarchy. While the square root of relative height ($ \sqrt{h/H} $) remained the dominant predictor (51.64%), the interaction terms collectively accounted for 7.92% of the total explained deviance, acting as the critical modifiers that fine-tune stem form predictions beyond the capacity of main effects.

## Discussion

This study demonstrates that the incorporation of interaction effects into generalized additive models substantially enhances stem taper prediction for Chinese fir, yielding results that outperform both conventional parametric equations and main-effect GAMs. While previous studies have established the viability of GAMs for taper modeling^[21,25,26]^, they predominantly relied on additive structures. Our results reveal a critical limitation of this approach, as the best-performing main-effect GAM was inferior to the benchmark Kozak-II model (Table 7). This seemingly counterintuitive finding underscores that the Kozak model, through its multiplicative structure and complex exponents, implicitly captures the interactive nature of stem tapering. A purely additive GAM fails to account for these synergies and consequently provides a biased representation of the underlying biological process. While traditional parametric models can also be refined by manually incorporating multiplicative or divisive interaction terms to improve performance, such modifications often rely on *a priori* assumptions or iterative trial and error to determine the correct functional form for a specific dataset. In contrast, the true power of the GAM framework was realized when we explicitly modeled these interactions using tensor product smooths, which allows for the automatic discovery of optimal interaction structures in a data-driven manner. The optimal interaction-inclusive model M17 reduced the cross-validation RMSE by 52.3% relative to the main-effect model M11. More importantly, it demonstrated robust predictive capacity by outperforming the rigorous Kozak-II parametric benchmark while offering superior residual homogeneity across all tree sizes (Fig. 4).

A key contribution of this study is the rigorous selection of the interaction structure based on predictive generalization rather than simple goodness of fit. Although the model with three two-way interactions (M18) achieved the best fit on the training data, the cross-validation results favored the simpler model with two interactions (M17). M17 yielded the lowest predictive errors in the validation phase (Table 7), suggesting that the additional interaction term between DBH and total height in M18 introduced redundancy or overfitting. This aligns with the principle of parsimony in statistical modeling^[40]^. The interaction between DBH and total height likely captured sample-specific noise rather than a general biological trend, whereas the interactions involving relative height are essential for defining the stem profile. Consequently, we recommend M17 as the optimal model since it strikes the best balance between model complexity and predictive robustness.

Furthermore, our comparison with machine learning approaches highlights a significant advantage of the interaction-inclusive GAM. While the artificial neural network (M20) achieved high accuracy comparable to the parametric benchmark, it functions as a black box where functional relationships are obscured^[28,29]^. In contrast, M17 not only outperformed both artificial neural networks and random forest models in predictive accuracy (Table 7) but also retained the inferential transparency of parametric models. The superiority of M17 over machine learning models can be attributed to its semi-parametric nature, which imposes a smoothness constraint consistent with biological growth, whereas models like random forests may overfit local data irregularities. Our findings suggest that a well-specified GAM can effectively bridge the gap between the interpretability of parametric equations and the flexibility of algorithmic modeling^[22]^, providing a transparent approach that is both accurate and scientifically insightful.

The statistical improvement observed in M17 aligns with the biological concept that tree stem form is not static but evolves with size, a phenomenon governed by biomechanical constraints^[6,8]^. The strong interaction between DBH and relative height, as visualized in Fig. 5a, aligns with the constant stress hypothesis^[7]^, which posits that trees allocate biomass to equalize bending stress along the stem. As illustrated in the interaction surface, trees with larger DBH exhibit a disproportionately pronounced basal flare compared to smaller trees. This reflects an adaptive response to increased mechanical load, as the stem must thicken at the base to prevent failure when crown size and wind drag increase with tree growth^[9]^. Furthermore, the interaction between total height and relative height shown in Fig. 5b indicates that taller trees modify their taper rates in the lower bole to maintain stability. As trees increase in total height, their slenderness ratio typically alters, subjecting the stem to greater wind-induced bending moments and leverage^[6,9]^. To counteract this increased wind loading and prevent structural failure, taller individuals must dynamically adjust their lower bole taper, distributing the mechanical stress more evenly across the lower stem profile^[7]^. By capturing these allometric shifts via tensor product smooths, the interaction-inclusive GAM effectively models the ontogenetic drift in stem form from paraboloid to neiloid shapes as trees mature without requiring the rigid and predefined inflection points used in variable-exponent models. The variable importance analysis confirmed that while the relative height is the dominant driver of taper, the interaction terms account for a non-negligible proportion of approximately 8% of the explained deviance (Fig. 6). This fraction represents the critical fine-tuning that distinguishes a standard model from a high-precision one.

For comparative purposes, the GAMs in this study utilized the same standard variables of diameter, height, and relative height as parametric approaches. However, it is well-established that stand attributes such as stand density and site index, along with crown metrics, also influence stem form^[27,32,33]^. This is particularly critical when extending our framework from structurally simple and even-aged plantations to natural forests. In such heterogeneous environments, uneven light distribution and complex competitive pressures often result in irregular crown development and more intricate allometric interactions affecting stem taper. The GAM framework is ideally suited to incorporate these additional variables as linear or smooth terms in future research, potentially through higher-order interactions, which could further refine predictive accuracy and ecological interpretability. Furthermore, the acquisition of such detailed stem form data has traditionally relied on labor-intensive destructive sampling, which limits sample size and geographic scope. The rapid advancement of remote sensing technologies, particularly terrestrial laser scanning (TLS), now offers a nondestructive alternative for quantifying 3D stem profiles with millimeter precision^[41,42]^. Integrating TLS-derived point clouds with the flexible and interaction-inclusive GAMs proposed here represents a promising frontier, enabling the development of dynamic and site-specific taper models on a significantly larger scale.

## Conclusions

This study demonstrates that explicitly modeling the nonlinear interactions between tree size and relative height is essential for resolving the systematic biases inherent in traditional taper models. By integrating tensor product interaction smooths, we developed a model for Chinese fir that not only outperforms leading parametric equations and black-box machine learning algorithms in predictive accuracy but also provides a transparent statistical framework to evaluate ontogenetic drift, such as the size-dependent basal flare. This approach effectively bridges the long-standing trade-off between algorithmic flexibility and biological interpretability, offering a robust and scientifically grounded tool for precision timber quantification and sustainable forest management.

## Data Availability

The datasets generated during and/or analyzed during the current study are available from the corresponding author upon reasonable request.

## References

[b1] 1Burkhart HE, Tomé M. 2012. Modeling Forest Trees and Stands. Dordrecht: Springer Netherlands. 458 pp. doi: 10.1007/978-90-481-3170-9

[2] (2022). Site quality and intensive early stand management practices affect growth dominance, structural complexity, and tree growth in ponderosa pine plantations. Forest Ecology and Management.

[3] (1969). Taper functions and their application in forest inventory. The Forestry Chronicle.

[4] (2024). Compatible taper and volume systems for *Larix olgensis* and *Larix kaempferi* in northeast China. European Journal of Forest Research.

[5] (1963). Stem form development of forest trees. Forest Science.

[b6] 6Niklas KJ. 1994. Plant allometry: The scaling of form and process. Chicago, USA: The University of Chicago Press. 412 pp. https://press.uchicago.edu/ucp/books/book/chicago/P/bo3629790.html

[7] (1986). Validity of constant-stress and elastic-instability principles of stem formation in *Pinus contorta* and *Trifolium pratense*. Annals of Botany.

[8] (2004). Growth and hydraulic (not mechanical) constraints govern the scaling of tree height and mass. Proceedings of the National Academy of Sciences of the United States of America.

[9] (1998). Ontogenetic changes in size, allometry, and mechanical design of tropical rain forest trees. American Journal of Botany.

[10] (1992). Variable-form taper functions for four Alberta tree species. Canadian Journal of Forest Research.

[11] (1988). A variable-exponent taper equation. Canadian Journal of Forest Research.

[12] (2004). My last words on taper equations. The Forestry Chronicle.

[13] (2021). Stem taper functions for white birch (*Betula platyphylla*) and costata birch (*Betula costata*) in the Xiaoxing'an Mountains, northeast China. Forestry: an International Journal of Forest Research.

[14] (2022). Incorporating stand density effects and regression techniques for stem taper modeling of a *Larix principis-rupprechtii* plantation. Frontiers in Plant Science.

[15] (2023). Taper equations for eight major forest tree species in flat land Ukraine. Forestry: an International Journal of Forest Research.

[16] (2024). Regional differences in stem form between southern and northern red spruce (*Picea rubens* Sarg.) populations. Forestry: an International Journal of Forest Research.

[17] (2025). Evaluation of taper measurement schemes for modeling stem profiles: a case study of two conifer species. Canadian Journal of Forest Research.

[18] (1976). Segmented polynomial regression applied to taper equations. Forest Science.

[19] (2021). Evolution, history, and use of stem taper equations: a review of their development, application, and implementation. Canadian Journal of Forest Research.

[20] (2022). Predicting the upper stem diameters and volume of a tropical dominant tree species. Journal of Forestry Research.

[21] (2011). Fitting forestry models using generalized additive models: a taper model example. Canadian Journal of Forest Research.

[22] (2023). Evaluating semi- and nonparametric regression algorithms in quantifying stem taper and volume with alternative test data selection strategies. Forestry: an International Journal of Forest Research.

[23] (1986). [Generalized additive models]: rejoinder. Statistical Science.

[b24] 24Wood SN. 2006. Generalized additive models: An introduction with R. Boca Raton: Chapman and Hall/CRC Press. 416 pp. doi: 10.1201/9781420010404

[25] (2021). Evaluation of four regression techniques for stem taper modeling of Dahurian larch (*Larix gmelinii*) in Northeastern China. Forest Ecology and Management.

[26] (2022). Evaluation of parametric and non-parametric stem taper modeling approaches: a case study for *Betula platyphylla* in Northeast China. Forest Ecology and Management.

[27] (2022). Analysis of various crown variables on stem form for *Cunninghamia lanceolata* based on ANN and taper function. Forest Ecology and Management.

[28] (2002). Illuminating the "black box" : a randomization approach for understanding variable contributions in artificial neural networks. Ecological Modelling.

[29] (2019). Stop explaining black box machine learning models for high stakes decisions and use interpretable models instead. Nature Machine Intelligence.

[b30] 30Wood SN. 2017. Generalized Additive Models: An Introduction with R. 2nd Edition. New York, USA: Chapman and Hall/CRC. 496 pp. doi: 10.1201/9781315370279

[b31] 31National Forestry and Grassland Administration. 2019. China forest resources report (2014-2018). Beijing, China: China Forestry Publishing House. 451 pp (in Chinese)

[32] (2016). Development of a stem taper equation and modelling the effect of stand density on taper for Chinese fir plantations in Southern China. PeerJ.

[33] (2021). Variable-exponent taper equation based on multilevel nonlinear mixed effect for Chinese fir in China. Forests.

[34] (1997). A study on taper equation. Scientia Silvae Sinicae.

[35] (2003). Modeling stem profiles for *Pinus densiflora* in Korea. Forest Ecology and Management.

[36] (2020). Stem taper modeling equation for Dahurian larch based on nonparametric regression methods. Journal of Nanjing Forestry University (Natural Sciences Edition).

[37] (2010). Neuralnet: training of neural networks. The R Journal.

[38] (2002). Classification and regression by randomForest. R News.

[39] (2024). Evaluating the relative importance of predictors in Generalized Additive Models using the *gam.hp* R package. Plant Diversity.

[b40] 40Weiskittel AR, Hann DW, Kershaw Jr JA, Vanclay JK. 2011. Forest Growth and Yield Modeling. New York, USA: John Wiley &amp; Sons. 415 pp. doi: 10.1002/9781119998518

[41] (2016). Terrestrial laser scanning in forest inventories. ISPRS Journal of Photogrammetry and Remote Sensing.

[42] (2023). Use of terrestrial laser scanning to obtain the stem diameters of *Larix olgensis* and construct compatible taper-volume equations. Trees.

